# A Case of Esophageal Perforation Secondary to Undiagnosed Eosinophilic Esophagitis

**DOI:** 10.7759/cureus.96164

**Published:** 2025-11-05

**Authors:** Reyleen R Loreto, Yesenia Gonzalez, Antoine Khoudari, Hazem Abugrara

**Affiliations:** 1 Internal Medicine, Baycare Health System, Tampa, USA

**Keywords:** eosinophilic esophagitis, esophageal perforation, gastrointestinal disorder, rare complications, undiagnosed inflammatory condition

## Abstract

Eosinophilic esophagitis (EoE) is a chronic, immune-mediated inflammatory disorder of the esophagus, increasingly recognized for its rising prevalence and potential for severe complications. While EoE typically presents with dysphagia, heartburn, and food impaction, it can remain asymptomatic until serious complications such as esophageal perforation occur. This paper highlights a case of a 38-year-old male with a past medical history of occasional gastroesophageal reflux disease (GERD) and reactive airway disease who presented with acute severe chest pain and dyspnea shortly after eating pizza. He was ultimately diagnosed with spontaneous esophageal perforation secondary to previously undiagnosed EoE. Diagnostic imaging revealed a significant mid-esophageal perforation accompanied by pneumomediastinum. Esophagogastroduodenoscopy (EGD) revealed a large esophageal perforation with a dilated esophagus and mucosal linear furrows, which were biopsied. Histopathological examination of the biopsies revealed an eosinophil count of up to 52 eosinophils per high-power field in the mid-esophagus. The patient’s management required multidisciplinary intervention, including endoscopic and surgical repair, endoluminal vacuum-assisted closure, and prolonged antimicrobial and nutritional support. These measures ultimately led to complete recovery without further complications. This case emphasizes the potential severity of undiagnosed EoE and highlights the importance of early diagnosis and intervention. Practitioners should maintain a high index of suspicion for EoE in patients with GERD or atypical esophageal symptoms to prevent potentially life-threatening outcomes.

## Introduction

Eosinophilic esophagitis (EoE) is a chronic, immune-mediated condition of the esophagus marked by dense eosinophilic infiltration of the mucosa [1,2]. Its development reflects a complex interaction between genetic susceptibility, environmental exposures early in life, and allergic responses-particularly to food antigens [1-4]. In predisposed individuals, contact with these allergens triggers the release of epithelial cytokines such as thymic stromal lymphopoietin (TSLP) and interleukin-33 (IL-33). These mediators activate type 2 helper T (Th2) lymphocytes, which in turn produce interleukins IL-4, IL-5, and IL-13. The resulting recruitment of eosinophils and mast cells sustains inflammation that, over time, leads to subepithelial fibrosis, tissue remodeling, and esophageal narrowing [4]. 

The estimated prevalence of EoE ranges from 34.4 cases per 100,000 individuals in Europe and North America, with a higher occurrence in adults than in children [1,5]. Its incidence continues to rise globally, reflecting both greater recognition and changing environmental or dietary influences. Adults commonly present with dysphagia, heartburn, or food impaction, while children may experience vomiting, abdominal discomfort, feeding difficulties, or occasional airway involvement [1,3,4,6]. 

Diagnosis requires evidence of esophageal dysfunction together with histologic confirmation of mucosal eosinophilia, defined as 15 or more eosinophils per high-power field on biopsy, after excluding other causes such as gastroesophageal reflux disease (GERD) [1,6,7]. Endoscopic findings often include linear furrows, whitish exudates, fragile mucosa, and concentric rings or strictures [6]. While treatments like proton pump inhibitors, swallowed topical corticosteroids, and dietary elimination methods can effectively manage inflammation and symptoms, complications might still occur in cases that are advanced or left untreated [6,8-10]. 

Although uncommon, esophageal perforation occurs in roughly 2% of EoE cases [3]. It generally develops in patients with unrecognized, long-standing disease complicated by inflammation and stricture formation, frequently following food impaction or endoscopic procedures. Perforation as an initial presentation is exceptionally rare [3,11]. This report describes an unusual case of esophageal perforation as the first manifestation of previously undiagnosed EoE, emphasizing the diagnostic challenges and the importance of maintaining clinical suspicion. 

## Case presentation

**Figure 1:**
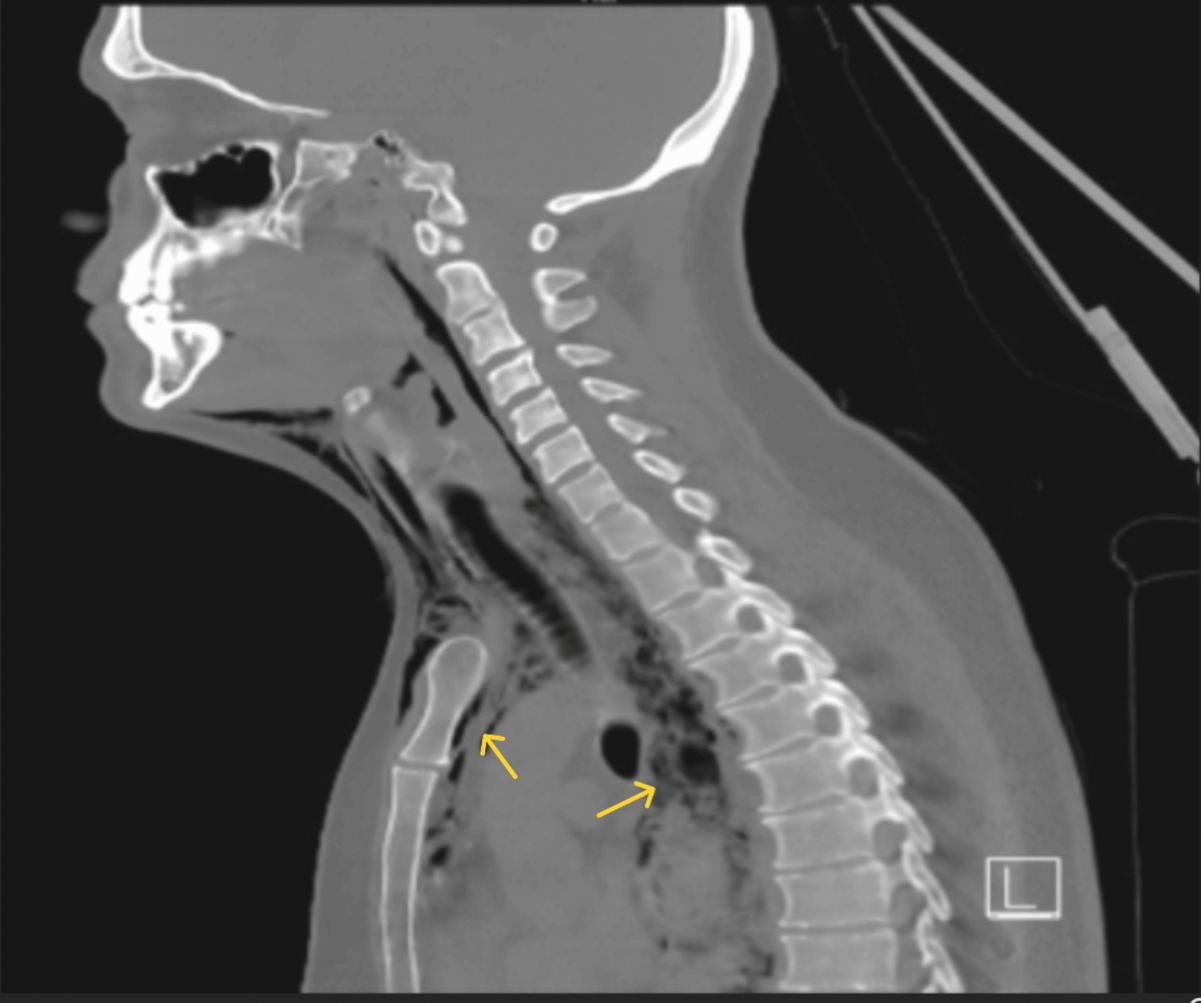
Sagittal section showing pneumomediastinum

**Figure 2:**
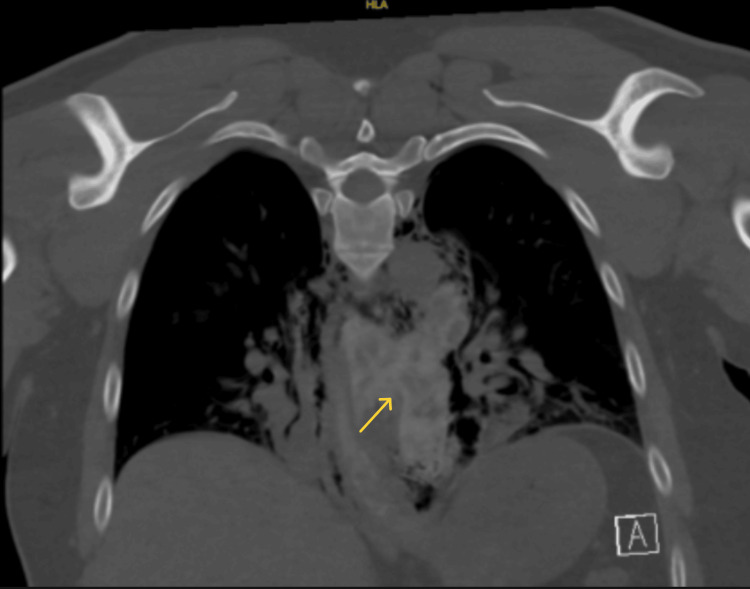
CT chest coronal view showing spillage of esophageal contents to the mediastinum

**Figure 3:**
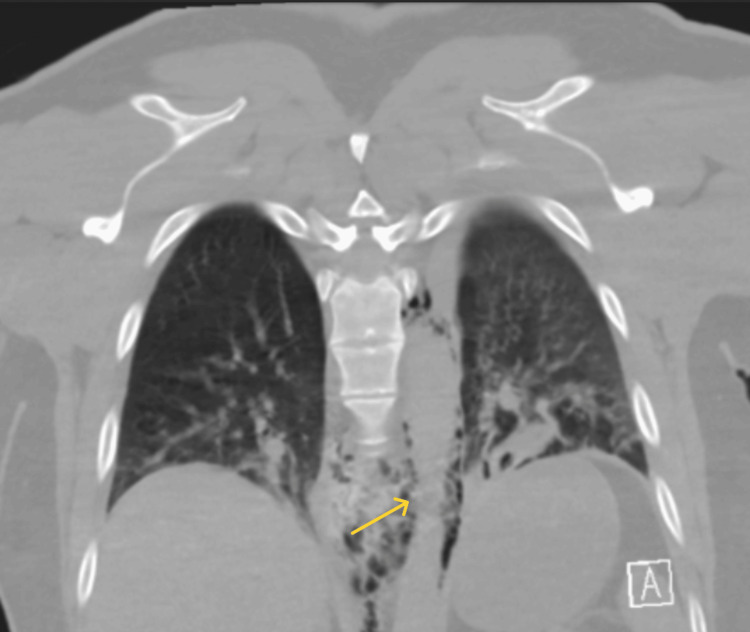
CT chest coronal view showing esophageal narrowing and perforation

A 38-year-old Caucasian male with past medical history of GERD managed with famotidine as needed, anxiety, and reactive airway disease presented with acute chest pain. Chest pain commenced suddenly, with a pain score of 10/10. The pain was crushing in character, located on the left side, and precipitated by eating thin-crust pizza, prompting an emergency medical service call from his spouse. The patient denied shortness of breath, nausea, vomiting, abdominal pain, fever, or any prior episodes of chest pain. He reported being a lifelong non-smoker and consuming alcohol infrequently, although he prefers spicy food. Upon hospital admission, the patient was hemodynamically stable and afebrile. Physical examination revealed the presence of subcutaneous air in the neck and anterior upper chest with diminished breath sounds in the bilateral lower lobes of the lung. He was also diaphoretic. No altered mental status, hyper- or hypotension, S4 or new murmur, stridor, hypoxia, or chest tenderness on palpation. CT scans revealed significant perforation in the left lateral wall of the mid-esophagus with contrast extravasation and suspected food debris in the mediastinum, along with small pleural effusions and ground-glass opacities at the lung bases (Figures 1, 2, 3). The initial differential diagnoses included esophageal perforation (Boerhaave’s syndrome), spontaneous pneumomediastinum, myocardial ischemia, pulmonary embolism, and acute aortic syndromes. 

**Figure 4:**
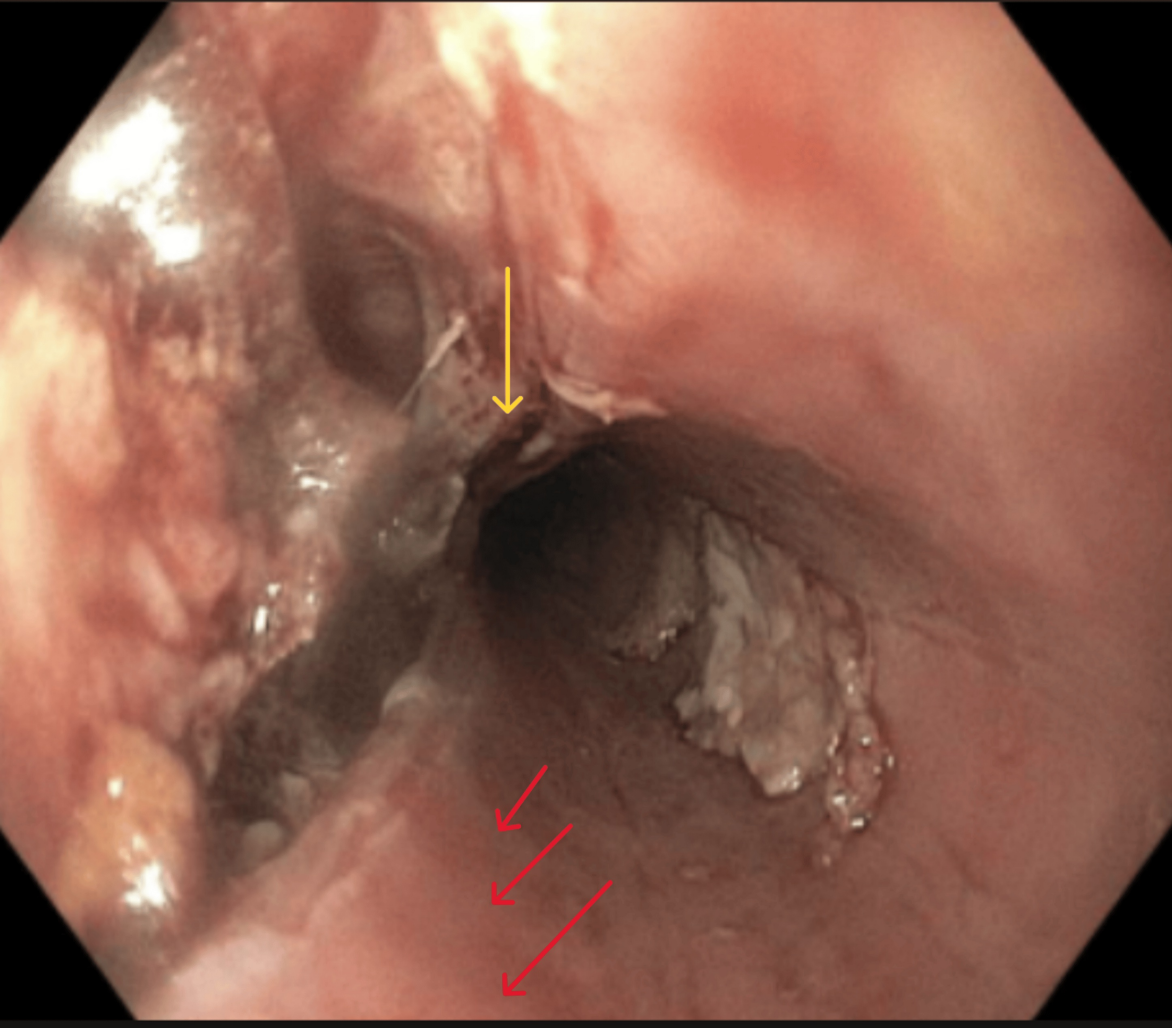
Esophageal perforation was observed (yellow arrow). The adjacent mucosa showed linear furrows (red arrows)

**Figure 5:**
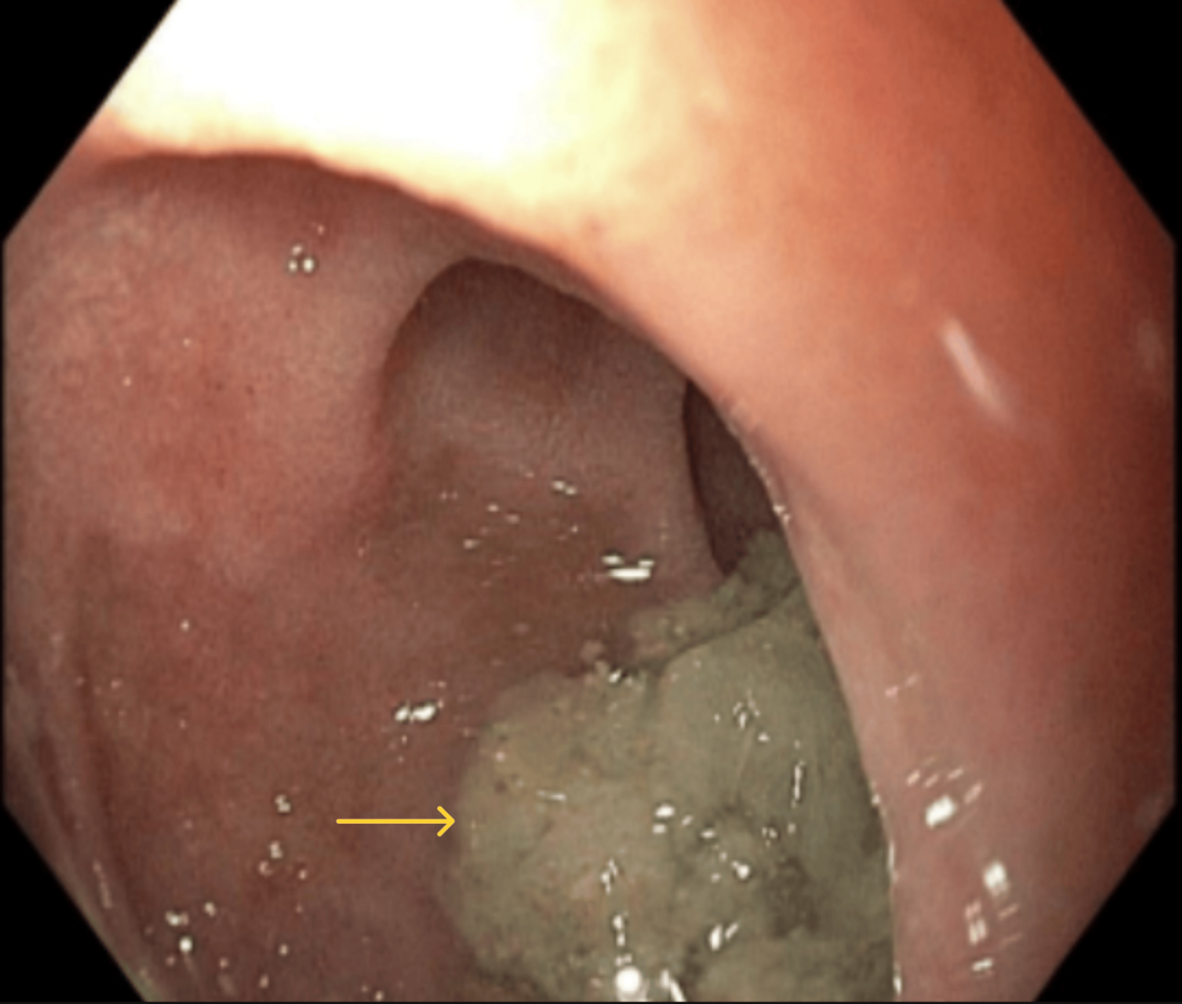
Food in the middle third of the esophagus

**Figure 6:**
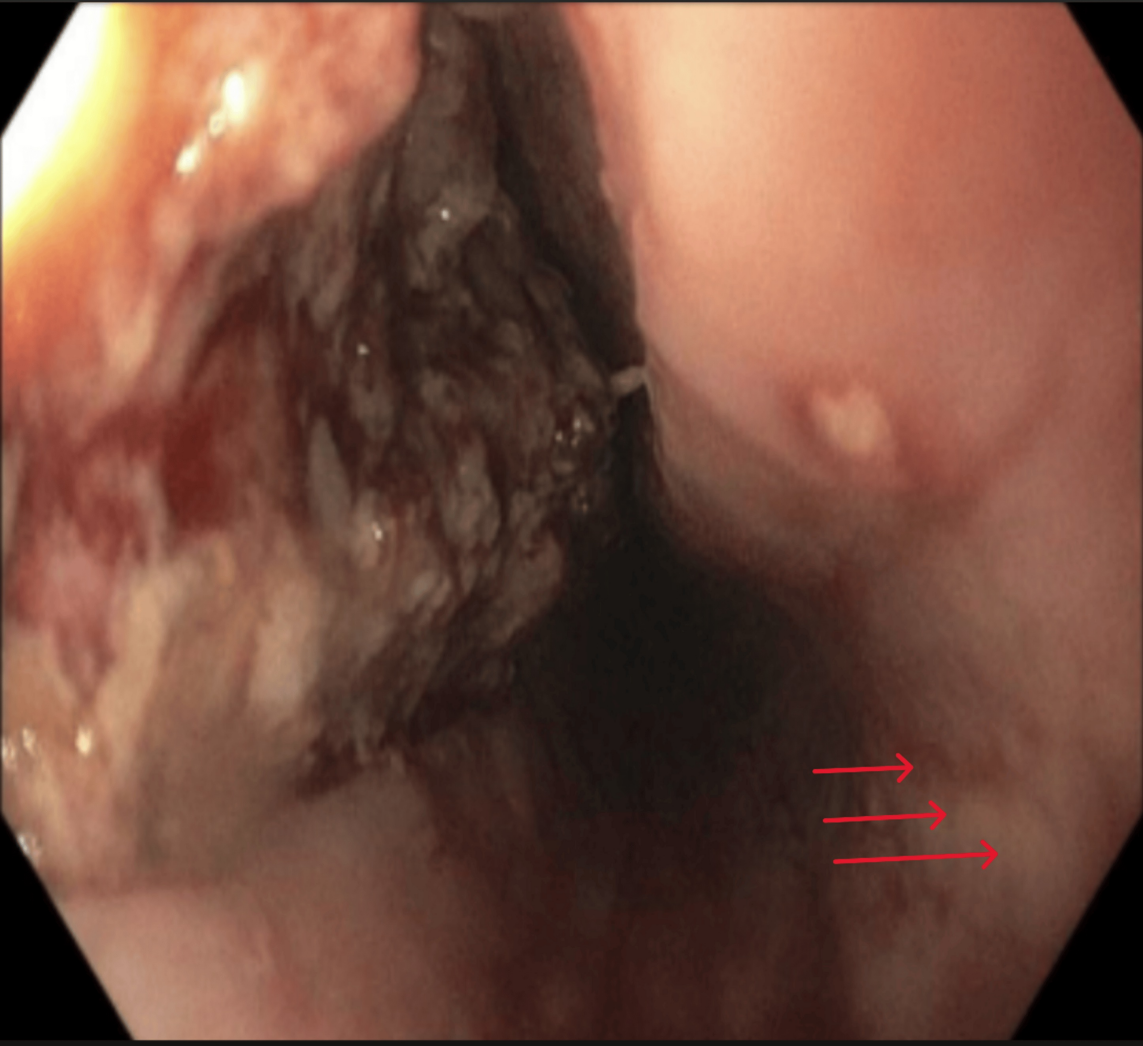
The food removal was successful. The adjacent mucosa showed linear furrows (red arrows)

Given the history of food ingestion immediately preceding symptom onset and the physical findings of subcutaneous emphysema, an esophageal source was highly suspected early in the evaluation period. Because of the life-threatening condition encountered, emergency consultations were made with minimally invasive surgery/bariatric surgery, thoracic surgery, and gastroenterology. An esophagogastroduodenoscopy (EGD) revealed a large esophageal perforation with dilated esophagus and mucosal linear furrows, which were biopsied (Figures 4, 5, 6). Initial attempts to place a fully covered esophageal stent were unsuccessful due to the stricture. Hemostatic clips were applied to partially close the perforation; however, some segments were too extensive to be adequately approximated or sealed with clips. The patient underwent robotic-assisted trans-hiatal drainage of a mediastinal abscess and mediastinitis with esophageal perforation repair and endoluminal vacuum-assisted closure (VAC) system placement. Pathology from mid-esophageal biopsies showed squamous mucosa with up to 52 eosinophils per high-power field, consistent with eosinophilic esophagitis.

Post-surgery, the patient was initiated on pantoprazole 40 mg IV twice daily. He required intensive care with mechanical ventilation and sedation, from which he was successfully weaned and extubated after two days. Total parenteral nutrition (TPN) was initiated immediately after surgery. Subsequently, a gastrojejunostomy (GJ) tube was placed to facilitate enteral feeding. Broad-spectrum antibiotics (meropenem) and antifungal therapy (fluconazole) were administered for one month due to the recurrence of mediastinal abscess and mediastinitis. Once the patient’s enteral nutrition via the GJ tube reached the goal rates, TPN was successfully discontinued, and the patient remained on nothing by mouth status throughout this period. During hospitalization, the patient developed bilateral pleural effusions, necessitating two left-sided thoracenteses. Analysis of the pleural fluid consistently revealed an exudative profile, with cytology negative for malignancy and cultures yielding no growth. 

**Figure 7:**
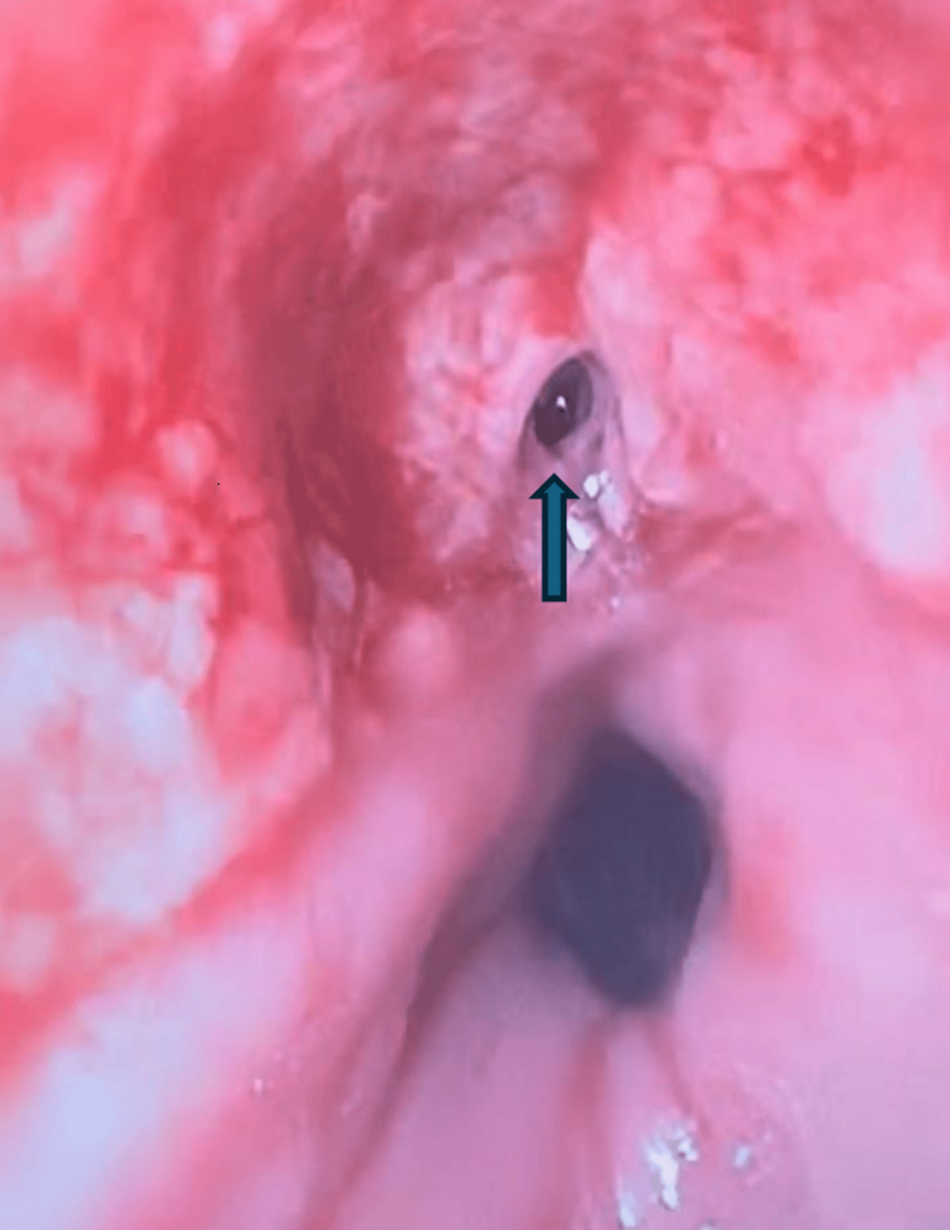
Fistula seen on esophagogastroduodenoscopy (EGD) which was closed using an amino acid composite sealant.

Multiple EGD procedures with transesophageal drainage and replacement of endoluminal VAC were performed. On the seventh EGD, performed for drainage of a residual intrathoracic abscess and exchange of the endoluminal VAC device, a fistulogram was also conducted under fluoroscopic guidance. This revealed a small persistent fistulous tract originating from the previous drain site, which drained back into the esophageal lumen with no evidence of an external leak (Figure 7). The previously noted esophageal perforation seemed well healed then. On the eighth and final EGD, the endoluminal VAC was removed and the fistula tract was closed using an amino acid composite sealant. Endoscopic evaluation confirmed a well-healed esophageal perforation with no visible drain, purulence, or ongoing extraluminal communication. 

Serial chest computed tomography (CT) scans were performed throughout the hospitalization period to monitor the healing of the perforation. A barium swallow study demonstrated no evidence of contrast extravasation, and chest CT with and without contrast confirmed the absence of any discrete esophageal perforation or active leakage. 

**Figure 8:**
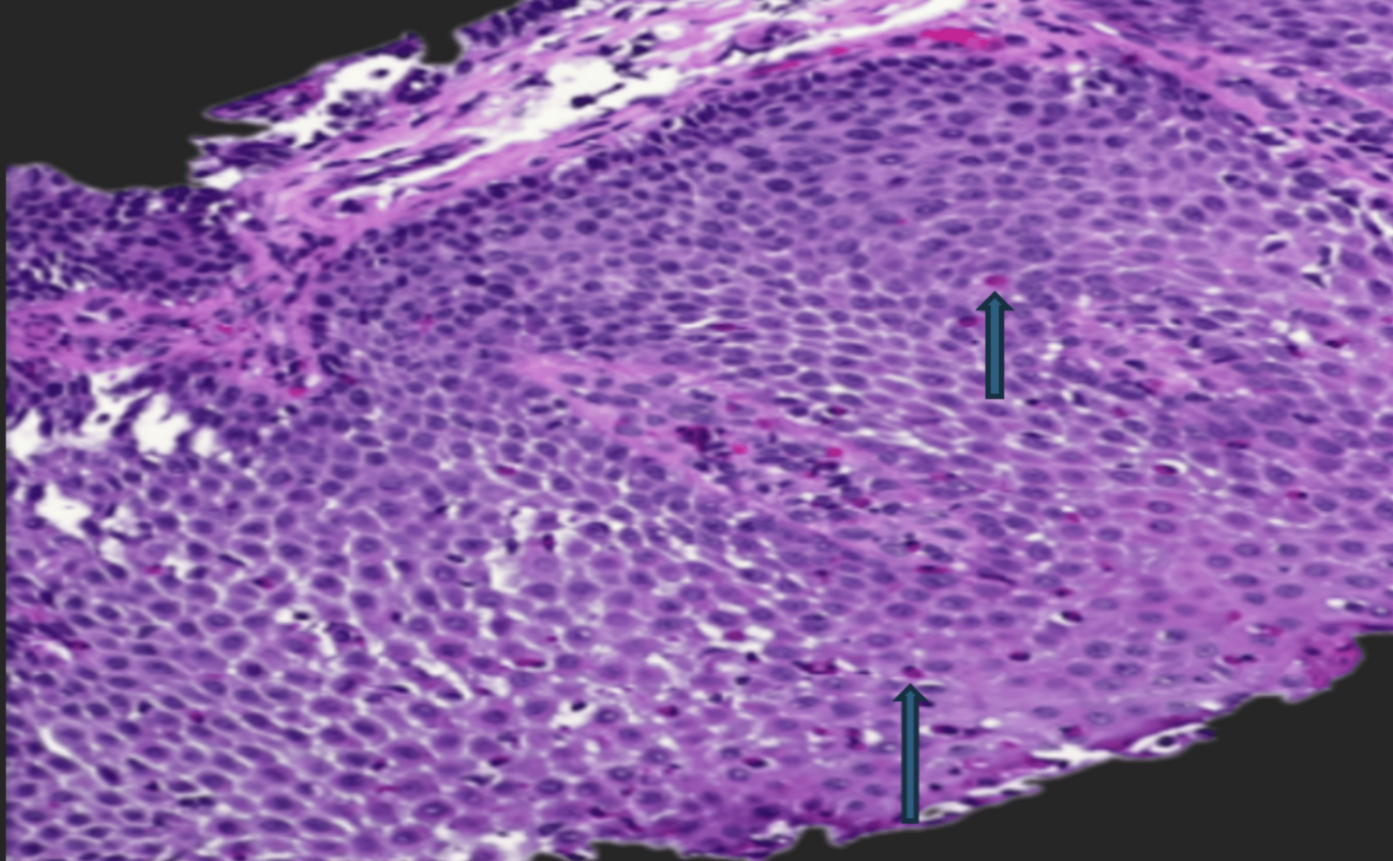
This slide demonstrates basal zone hyperplasia with increased eosinophils predominantly located in the mid and upper levels of the mucosa

**Figure 9:**
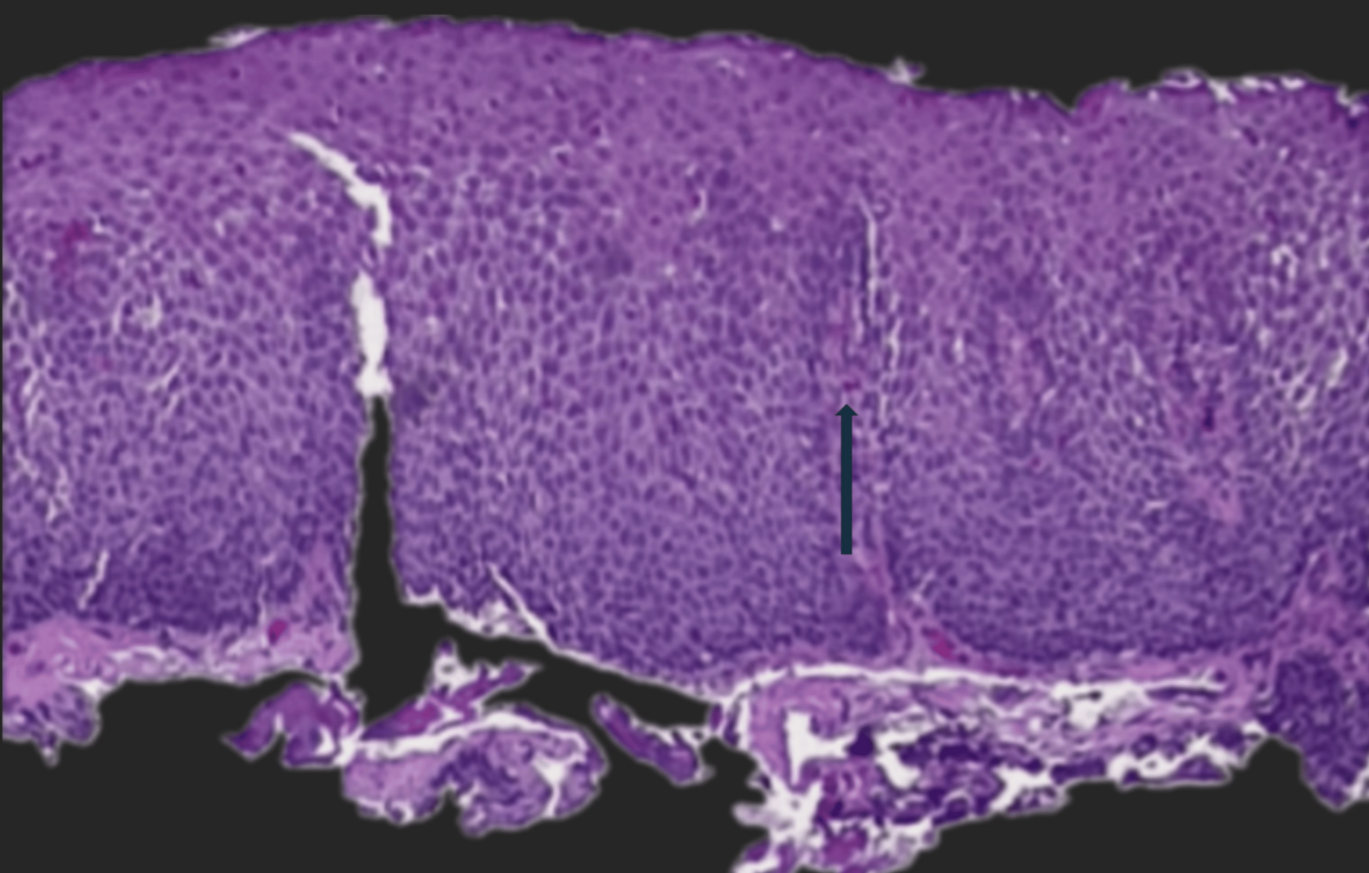
Basal zone hyperplasia was again observed, with increased eosinophils mainly in the mid and upper layers of the mucosa

The patient denied abdominal pain, nausea, or vomiting prior to discharge. During follow-up with the Surgery service, he remained on bolus tube feeding and was transitioned to a liquid diet after a repeat esophagogram demonstrated no residual esophageal leak. Two weeks post-discharge, a repeat EGD performed by Surgery showed a well-healed site from the prior perforation with mild narrowing of the distal esophagus. The GJ tube was replaced with a gastrostomy tube. Biopsy from the distal esophagus was negative for eosinophilic infiltration. As he tolerated advancement to a soft diet without abdominal pain, dysphagia, or retrosternal burning, the gastrostomy tube was subsequently removed. He then self-discontinued pantoprazole. The patient was referred to Gastroenterology to assess the possible need for esophageal dilation. A repeat EGD performed three and a half months after the initial diagnosis confirmed persistent eosinophilic esophagitis. Endoscopic findings revealed longitudinal furrows, severe congestion throughout the esophagus, and a single non-bleeding gastric ulcer with a clean base. Biopsies from the proximal and distal esophagus demonstrated reactive squamous mucosa with up to 55 eosinophils per high-power field (hpf) proximally and up to 110 eosinophils/hpf distally, with focal ulceration in both segments (Figures 8, 9). Gastric biopsies showed chronic gastritis without evidence of dysplasia, malignancy, or Helicobacter pylori infection on Giemsa staining. He subsequently started dupilumab 300 mg subcutaneous injection once weekly, and pantoprazole 40mg was continued once daily. The patient has remained under regular follow-up with Gastroenterology. 

## Discussion

EoE is an increasingly recognized immune-mediated esophageal disease, defined by symptoms of physiological dysfunction, and marked infiltration with eosinophils within the esophageal mucosa (≥15 eosinophils per high-power field on histology) [1,6,7]. The condition was initially described in children but is now well recognized in adults, with a significant increase in prevalence over the past two decades. This is attributed to more disease awareness and improved diagnostic criteria [2]. Current estimates suggest a prevalence of 0.5-1 per 1,000 individuals in Western populations, with adult males being disproportionately affected [2,5,12]. 

The clinical presentation of EoE in adults can be subtle and can involve intermittent dysphagia, food impaction, or chest discomfort [1,3,4,6]. Due to the subtlety of the symptoms, patients may become accustomed to or adapt behaviorally by avoiding certain foods or increasing mastication time, leading to delays in diagnosis [1,6]. Retrospective studies suggest that adult patients may experience symptoms for several years before diagnosis, with a median diagnostic delay of over six years [6]. This case illustrates such a delay: our patient denied a history of intermittent dysphagia and food impaction, with his only symptoms being occasional GERD; he had never undergone formal gastrointestinal evaluation prior to his acute presentation with spontaneous esophageal perforation. 

Spontaneous esophageal perforation (Boerhaave syndrome) is a life-threatening condition characterized by a full-thickness rupture of the esophageal wall following a sudden increase in intraluminal pressure. The clinical presentation can include severe chest pain that radiates to the back and may be associated with subcutaneous emphysema, as seen in our patient [13]. It carries a high morbidity and mortality rate, especially when diagnosis and management are delayed [14]. While Boerhaave syndrome can be seen in healthy individuals, as in the case of excessive emesis, structural and inflammatory esophageal diseases such as EoE may significantly increase susceptibility by weakening the esophageal wall. The most common site of perforation is the left posterolateral wall of the distal third of the esophagus, just above the gastroesophageal junction. This is due to an anatomical area of weakness in the esophageal wall between the oblique muscle fibers [11,13,15]. This is consistent with the esophageal perforation in our patient, which occurred in the left lateral wall of the mid-esophagus. 

In the context of EoE, chronic eosinophilic inflammation leads to progressive tissue remodeling, lamina propria fibrosis, smooth muscle hypertrophy, and angiogenesis [16]. These changes reduce esophageal compliance and increase tissue fragility, making the esophagus more susceptible to injury from mechanical forces [2]. Endoscopic findings that suggest EoE, such as rings, linear furrows, white plaques, and mucosal fragility, are present in approximately 50-80% of cases [6]. However, a normal-appearing esophagus does not exclude the diagnosis, emphasizing the importance of biopsy in patients with suggestive symptoms of the disease. Our patient had faint linear furrows in proximity to the perforation, which were biopsied.  

Although food impaction is the most common acute complication of EoE, esophageal perforation remains a rare but serious event. A systematic review by Dellon et al. identified 55 cases of esophageal perforation associated with EoE; in over two-thirds of these cases, EoE was diagnosed only after the complication occurred, just as in our case [7]. Most of these patients were adult males with no serious past medical history, highlighting the potential for EoE to remain asymptomatic until an event reveals its presence in the esophagus. 

The management of esophageal perforation is multifaceted and depends on several factors, including the location and size of the perforation, time to presentation, degree of contamination, and patient stability. Imaging, typically with contrast-enhanced CT or water-soluble esophagography, is essential for confirming the diagnosis. In our patient, imaging revealed extensive pneumomediastinum and partially visualized subcutaneous emphysema within the soft tissues of the neck. He first underwent endoscopic repair; however, owing to his perforation size, he eventually underwent surgical repair and broad-spectrum antimicrobial therapy. During the initial endoscopy, faint linear furrows were observed near the perforation, prompting a biopsy, which revealed histological evidence of EoE.

The definitive diagnosis of EoE is histology [6,7]. Initial biopsy revealed 52 eosinophils per high-power field (officially diagnosis can be made with at least six biopsies taken between the proximal and distal esophagus). In our case, esophageal mucosal biopsies performed later revealed dense eosinophilic infiltration, with peak counts exceeding 110 eosinophils per high-power field (eos/hpf), consistent with EoE. According to the updated 2018 Appraisal of Guidelines for Research and Evaluation (AGREE) consensus recommendations, EoE is diagnosed when eosinophilic inflammation is markedly confined to the esophagus and other potential etiologies of esophageal eosinophilia are not identified [17]. 

The Eosinophilic Esophagitis Histology Scoring System (EoEHSS) objectively evaluates the grade and stage of multiple histopathologic features and has been shown to outperform peak eosinophil count in distinguishing treated from untreated disease [18]. For this patient, the EoEHSS rating was 0.25, consistent with mild disease (Table 1). 

**Table 1:** Eosinophilic Esophagitis Histology Scoring System (EoEHSS) rating. A grade and stage score closer to 1 indicates more severe and extensive disease, respectively. This patient’s EoEHSS score of 0.25 corresponds to mild eosinophilic esophagitis.

The Eosinophilic Esophagitis Histology Scoring System (EoEHSS)	
Esophageal inflammation	2
Basal zone hyperplasia	2
Eosinophil abscess	0
Eosinophil surface layering	0
Dilated intercellular spaces	2
Surface epithelial alteration	0
Dyskeratotic epithelial cells	0
Lamina propria fibrosis	0
EoEHSS rating	0.25

On the other hand, the EoE-Endoscopic Reference Score (ERS) is a validated tool used to assess disease severity. It evaluates endoscopic features such as rings, exudates, furrows, edema, and stricture to grade the degree of severity [6]. Although this scoring system was not yet validated as outcome measures with mixed correlation to eosinophil counts, it correlates better with the disease severity in this patient (Table 2).

**Table 2:** EoE Endoscopic Reference Score (EoE-ERS) *These findings indicate active inflammatory changes alongside fibrostenotic remodeling, consistent with advanced eosinophilic esophagitis.

The Eosinophilic Esophagitis Endoscopic Reference Score	
Finding	Grade
Edema	Grade 1 (decreased vascular markings)
Rings	Grade 3 (severe)
Exudate	Grade 2 (severe)
Furrows	Grade 2 (severe)
Stricture	Grade 1 (present)
Total score	9*

Discrepancies between the EoEHSS and the EoE-ERS are not unusual in clinical practice. These variations may result from the imperfect correlation between histologic and endoscopic findings, the patchy distribution of inflammation in EoE, sampling variability, and the distinct parameters each scoring system is designed to assess in evaluating disease activity. Despite these inconsistencies, histologic confirmation remains the cornerstone for both diagnosis and ongoing assessment of EoE, irrespective of the endoscopic appearance [19]. 

The management of EoE focuses on reducing esophageal eosinophilia, improving symptoms, and preventing long-term complications, such as strictures, food impaction, and, as in this case, perforation. The mainstays of treatment include proton pump inhibitors (PPIs), topical corticosteroids, and dietary elimination therapy. PPIs may be effective in 30-50% of patients, independent of acid suppression, owing to their anti-inflammatory properties [8]. Topical corticosteroids, such as swallowed fluticasone or budesonide, are the first-line pharmacologic therapy for most patients and have demonstrated both histologic and symptomatic efficacy in multiple randomized controlled trials [9,20]. Our patient was initiated on pantoprazole twice a day, with a favorable clinical response. 

Dietary therapy is another evidence-based approach to the management of EoE. The six-food elimination diet (SFED), which involves the restriction of dairy, wheat, eggs, soy, nuts, and seafood, has demonstrated histologic remission rates of 70-80% in prospective studies [10]. More recently, step-up elimination protocols have been proposed to improve adherence and minimize dietary burden while still achieving remission [21]. Our patient forgone eating foods like steak and had been eating smaller bites of food and chewing well.

Recent studies have shown that biologic agents targeting the type 2 inflammatory pathway, such as dupilumab, administered as a weekly subcutaneous injection, can improve both clinical symptoms and esophageal histology in patients with EoE [22]. Dupilumab is currently approved for the treatment of EoE. In contrast, newer therapies aimed at depleting eosinophils, such as the monoclonal antibody benralizumab, have not demonstrated consistent improvement in dysphagia symptoms [23]. In our patient, treatment with dupilumab 300 mg subcutaneous injection once weekly was initiated.

Therefore, long-term monitoring of EoE is essential. Even in the absence of symptoms, subclinical inflammation can persist and contribute to the progression of fibro-stenotic disease. Repeat endoscopy with biopsy is typically recommended eight to 12 weeks after initiating therapy to assess histologic remission.

EoE should be considered in any adult presenting with chronic dysphagia or food impaction, even in the absence of a relevant medical history. Second, although rare, spontaneous esophageal perforation can be the initial manifestation of EoE and should prompt evaluation for underlying mucosal disease. Third, a multidisciplinary approach, including surgical intervention, histopathological confirmation, allergist referral, and dietary/nutritional guidance, is crucial for the acute and long-term management of such patients.

## Conclusions

This case highlights a rare presentation of esophageal perforation secondary to EoE precipitated by food ingestion. Prompt recognition of subcutaneous emphysema and rapid imaging facilitated early diagnosis of perforation, allowing timely multidisciplinary intervention. Definitive management requires combined endoscopic, surgical, and endoluminal VAC approaches, along with prolonged antimicrobial therapy and nutritional support.

Early identification of EoE is critical, as untreated disease can lead to progressive esophageal remodeling, stricture formation, and life-threatening complications, such as spontaneous perforation. In this patient, the diagnosis of EoE during acute management provided an opportunity for targeted therapy to reduce the risk of recurrence of the disease. Long-term follow-up revealed persistent EoE, emphasizing the need for continued gastrointestinal surveillance and medical treatment. This case underscores the importance of maintaining a high index of suspicion for EoE in patients with suggestive symptoms, enabling early intervention to prevent severe complications and to optimize patient outcomes.
